# Association Between Assistance With Medicaid Enrollment and Use of Health Care After Incarceration Among Adults With a History of Substance Use

**DOI:** 10.1001/jamanetworkopen.2021.42688

**Published:** 2022-01-07

**Authors:** Marguerite E. Burns, Steven Cook, Lars M. Brown, Laura Dague, Steve Tyska, Karla Hernandez Romero, Cici McNamara, Ryan P. Westergaard

**Affiliations:** 1 Department of Population Health Sciences, University of Wisconsin School of Medicine and Public Health, Madison; 2 Institute for Research on Poverty, University of Wisconsin–Madison, Madison; 3 Wisconsin Department of Corrections, Madison; 4 The Bush School of Government and Public Service, Texas A&M University, College Station; 5 Division of Medicaid Services, Wisconsin Department of Health Services, Madison; 6 Department of Economics, University of Wisconsin–Madison, Madison; 7 Department of Medicine, University of Wisconsin School of Medicine and Public Health, Madison

## Abstract

**Importance:**

The transition from prison to community is characterized by elevated morbidity and mortality, particularly owing to drug overdose. However, most formerly incarcerated adults with substance use disorders do not use any health care, including treatment for substance use disorders, during the initial months after incarceration.

**Objective:**

To evaluate whether a prerelease Medicaid enrollment assistance program is associated with increased health care use within 30 days after release from prison.

**Design, Setting, and Participants:**

This retrospective cohort study included 16 307 adults aged 19 to 64 years with a history of substance use who were released from state prison between April 1, 2014, and December 31, 2016. The Wisconsin Department of Corrections implemented prerelease Medicaid enrollment assistance in January 2015. Statistical analysis was performed from January 1 to August 31, 2021.

**Exposure:**

A statewide Medicaid prerelease enrollment assistance program.

**Main Outcomes and Measures:**

The main outcome was Medicaid-reimbursed health care, associated with substance use disorders and for any cause, within 30 days of prison release, including outpatient, emergency department, and inpatient care. Mean outcomes were compared for those released before and after implementation of prerelease Medicaid enrollment assistance using an intention-to-treat analysis and person-level data from the Wisconsin Department of Corrections and Medicaid.

**Results:**

The sample included 16 307 individuals with 18 265 eligible releases (men accounted for 16 320 of 18 265 total releases, and 6213 of 18 265 releases were among Black individuals; mean [SD] age at release, 35.5 [10.7] years). The likelihood of outpatient care use within 30 days of release increased after implementation of enrollment assistance relative to baseline by 7.7 percentage points for any visit (95% CI, 6.4-8.9 percentage points; *P* < .001), by 0.7 percentage points for an opioid use disorder visit (95% CI, 0.4-1.0 percentage points; *P* < .001), by 1.0 percentage point for any substance use disorder visit (95% CI, 0.5-1.6 percentage points; *P* < .001), and by 0.4 percentage points for receipt of medication for opioid use disorder (95% CI, 0.2-0.6 percentage points; *P* < .001). There was no significant change in use of the emergency department (0.7 percentage points [95% CI, –0.15 to 1.4 percentage points]). The probability of an inpatient stay increased by 0.4 percentage points (95% CI, 0.03-0.7 percentage points; *P* = .03).

**Conclusions and Relevance:**

The results of this cohort study suggest that prerelease Medicaid enrollment assistance was associated with increased use of outpatient health care after incarceration and highlights the value of making this assistance universally available within correctional settings. More tailored interventions may be needed to increase the receipt of treatment for substance use disorders.

## Key Points

**Question:**

Is prison-based Medicaid enrollment assistance associated with increased use of health care within 30 days of prison release among adults with a history of substance use?

**Findings:**

In this cohort study of 16 307 individuals, the availability of prerelease Medicaid enrollment assistance was associated with large absolute increases in the likelihood of any outpatient visit and small or no absolute increases for substance use–associated and hospital-based care use.

**Meaning:**

This study found that the addition of Medicaid enrollment assistance to discharge planning in correctional settings was associated with increased outpatient health care use for individuals with substance use disorders during the immediate reentry period.

## Teaser

This cohort study evaluates whether a prerelease Medicaid enrollment assistance program was associated with increased health care use within 30 days after release from prison.

## Introduction

Substance use disorders (SUDs) are highly prevalent and undertreated among the 1.2 million individuals incarcerated in state prisons.^1,2^ Approximately 58% of adults incarcerated in state prisons meet the *Diagnostic and Statistical Manual of Mental Disorders* (Fourth Edition) criteria^3^ for drug dependence or abuse.^4^ However, only 28% of those individuals receive any form of drug treatment, and less than 0.5% receive medication treatment, while incarcerated. On release from prison, the period of reentry to the community is characterized by a high incidence of morbidity^5^ and elevated rates of mortality, particularly owing to drug overdose.^6,7,8^ Treatment of SUDs during this transition is associated with a reduced risk of relapse and overdose.^9,10^ However, most formerly incarcerated adults with an SUD do not receive any treatment in the initial months or in the year after release.^5^

Facilitating access to health care during the reentry period is a national priority, as articulated in the Medicaid reentry provision of the 2018 Substance Use-Disorder Prevention that Promotes Opioid Recovery and Treatment (SUPPORT) for Patients and Communities Act^11^ and the proposed Medicaid Reentry Act.^12^ Both policies aim to ensure that eligible individuals enroll in Medicaid before they exit prison or jail, thereby reducing financial barriers to receiving substance use treatment and health care more generally. For adults with SUDs, improved access to the full range of health services is important to address the relatively high prevalence of comorbid mental and physical illnesses that accompany addiction disorders.^13^ Furthermore, more frequent contact with health care professionals may facilitate initiation of treatment for SUDs^14^ through increased likelihood of diagnosis, the development of a trusting patient-clinician relationship, and opportunities for the patient to cultivate a readiness to engage in treatment.^15^ It is unknown, however, whether prison-based Medicaid enrollment assistance is associated with increased substance use treatment, or health care use more generally, for adults with a history of substance use in the immediate period after incarceration.

Several streams of research support our hypothesis that prerelease Medicaid enrollment assistance is associated with greater postincarceration use of health care, including substance use treatment. First, the availability of prison-based Medicaid enrollment assistance is associated with increased Medicaid enrollment.^16,17^ Second, among low-income adults, expanded Medicaid availability is associated with improved access to SUD treatment.^18,19,20,21,22,23,24,25,26,27^ Having Medicaid coverage is associated with increased outpatient,^28^ emergency department (ED),^29^ and inpatient visits.^30^ Finally, when tailored to prisoners with serious mental illness, Medicaid enrollment and referral assistance were associated with increased use of outpatient mental health care within 3 and 12 months of release^31,32^ and with treatment for substance use within 3 months of release.^33^ We examined the association between a newly available, statewide prison-based Medicaid enrollment assistance program and postincarceration outpatient, inpatient, and ED care use within 30 days of prison release among adults with a history of substance use.

## Methods

### Sample

The sample included adults aged 19 to 64 years with a history of substance use who were released from a Wisconsin state prison between April 1, 2014, and December 31, 2016, after an incarceration period of at least 31 days. For these individuals, we included all 18 265 releases from incarceration episodes that met this minimum duration criterion (eTable 1 in the Supplement). This study was determined exempt and participant consent was waived by the University of Wisconsin’s institutional review board because the study was determined to be secondary research that did not require consent. This study followed the Strengthening the Reporting of Observational Studies in Epidemiology (STROBE) reporting guideline.

### Study Design

In this retrospective cohort study, we leveraged a natural experiment to evaluate the association between a prerelease enrollment assistance program and postrelease health care use for adults with a history of substance use. In January 2015, the Wisconsin Department of Corrections (DOC) introduced a prerelease Medicaid enrollment assistance program for all adults under the supervision of the state’s Division of Adult Institutions in state correctional facilities. Discharge planning staff provide guidance on how to apply for Medicaid, after which individuals may call an eligibility case worker to apply. In addition, 5 facilities share 3 paralegal benefits specialists to assist with the program (eAppendix 1 in the Supplement). After the program’s implementation, Medicaid enrollment in the month of release increased by 25 percentage points, an increase that was associated with completion of applications before release from prison (eFigure 1 in the Supplement).^16^ At the time of the program’s implementation, Medicaid coverage in Wisconsin was available to adults with incomes below 100% of the federal poverty level, thus encompassing most adults leaving prison.^34^

### Data Sources

We linked person-level data from several Wisconsin agencies, the DOC, the Division of Medicaid Services, the State Lab of Hygiene, and the Electronic Data Surveillance System in the Institute for Research on Poverty’s Wisconsin Administrative Data Core.^35^ Data were matched using the last 4 digits of the participants’ Social Security numbers, DOC identification numbers, names, dates of birth, and other characteristics. We used fuzzy matching methods^36^ to account for name variants, data entry errors, or quality issues.

We considered individuals to have a history of substance use if they met any of the following criteria: self-reported opioid use, living with or at risk of hepatitis C virus (HCV), or a highly probable need for SUD treatment. We obtained the determination of treatment need and self-reported opioid use from the DOC’s risk and needs assessment tool, the Correctional Offender Management Profiling for Alternative Sanctions (COMPAS) tool.^37,38^ The COMPAS tool assesses substance use, history of substance use, and treatment for substance use. A proprietary algorithm converts individuals’ responses into a score that is intended to reflect their need for treatment as unlikely, probable, or highly probable.^23,24^ We defined persons living with, or at risk of, HCV as those who received a prescription medication for HCV while incarcerated, received a referral to receive an HCV test on prison admission, or ever had a positive antibody test result for HCV before release from prison (eAppendix 2 and eFigure 2 in the Supplement).

### Outcomes

We examined 4 binary outpatient care outcomes (eAppendix 3 in the Supplement): any outpatient visit, a visit associated with any SUD, a visit associated with opioid use disorder (OUD), and receipt of medication for OUD. Medication for OUD refers to a claim or Healthcare Common Procedure Coding System code for buprenorphine, naltrexone (oral), injectable naltrexone, buprenorphine-naloxone, or methadone. We assessed the probability of any ED visit or an inpatient admission for any cause or associated with a drug overdose. We observed only health care use that was paid by Medicaid. We measured all outcomes within 30 days of the individual’s release from prison; individuals with addiction disorders are at high risk of overdose during these initial weeks, highlighting the importance of promptly connecting them to care.^8^

### Covariates

All regression models included the following demographic characteristics: age, sex, educational level (ie, less than high school or General Educational Development Certification [GED] or at least high school or GED), marital status (ie, married, single, or other), race and ethnicity, and whether the county of conviction is part of a metropolitan statistical area. Race and ethnicity were included as covariates in the model because they may be associated with health care use and were measured in 3 categories as recorded by staff: Black, White, and other (which includes American Indian or Alaska Native, Asian or Pacific Islander, and unknown). County of conviction served as a proxy for county of release.^39^ Additional covariates included the security level of the release facility (ie, jail, minimum security, medium security, medium and maximum security, or maximum security) and the type of release (ie, supervised or unsupervised).

### Statistical Analysis

Statistical analysis was performed from January 1 to August 31, 2021. We tested the equivalence of mean characteristics for individuals released before and after implementation of enrollment assistance using normalized differences. The normalized difference is the difference in the mean of the covariate across the 2 study periods scaled by a measure of their SDs; it is useful in our large-sample context because it is relatively insensitive to sample size.^40^ Conventionally, normalized differences of 0.25 and smaller indicate good balance between the covariate mean values across groups.^41^ For the 2 variables for which data were missing, we created a “missing” category.

We compared the equivalence of unadjusted outcomes after implementation of enrollment assistance relative to the baseline period using the *t* test. We then implemented an intention-to-treat analysis using an ordinary least-squares estimator. The intention-to-treat estimate reflects the mean difference in Medicaid-paid health care use among individuals released after implementation of the enrollment assistance program compared with those released during the baseline period. This analysis provides an unbiased estimate of the association between exposure to the enrollment assistance program and postrelease health care use under the assumption that there were no confounding events or sample changes. We examined the plausibility of this assumption in 3 ways. First, we calculated the normalized differences in sample characteristics across study periods to test for changes in sample composition. Second, we confirmed with DOC colleagues that there were no significant DOC policy or programmatic changes associated with the likelihood of admission or release. Third, we plotted the unadjusted outcome trends to explore shifts or discontinuities that may indicate a confounding event.

We evaluated all outcomes with the full sample and conducted subgroup analyses for the most frequent outcome (any outpatient visit). Regression models included the covariates already described; SEs were clustered at the person level (eAppendix 4 in the Supplement). We used Stata, version 16 (StataCorp)^42^ to conduct the analyses. All *P* values were from 2-sided tests and, results were deemed statistically significant at *P* < .05.

### Sensitivity Analysis

Our main analyses included all releases because we sought to understand the association of enrollment assistance with postrelease care use for a typical cross-section of releases, which always includes some mix of first, second, and Nth releases. However, recognizing the possibility of a dose-response association between exposure to enrollment assistance, the likelihood of Medicaid enrollment, and subsequent postrelease health care use, we reestimated our models for first releases only. We estimated a specification with release facility fixed effects. Finally, we compared the expected differences between the baseline period and the enrollment assistance program for programs with or without a benefits specialist present to explore potential variation by program model.

## Results

The sample included 16307 individuals with 18265 eligible releases. The unit of analysis is the prison release. Men accounted for 16320 of 18265 releases [89.4%], with a mean [SD] age at release of 35.5 [10.7] years (Table 1). Of 18 265 releases, 6213 (34.0%) were among Black individuals, while White individuals accounted for 10 969 releases (60.1%), and 1083 releases (5.9%) were among individuals of other races and ethnicities. More than two-thirds of releases (12 465 [68.2%]) were among individuals who had a high school diploma or GED, and 15 921 (87.2%) were among single individuals. Missingness in the educational level variable (842 [4.6%]) and in the rurality of the conviction county variable (80 [0.4%]) was modest and constant over time. The magnitudes of all normalized differences in the covariate mean values fell below the 0.25 threshold, indicating comparability across study periods.^41^

**Table 1.** Characteristics of Individuals Aged 19 to 64 Years Released From Wisconsin State Prison With a History of Substance Use, From April 2014 to December 2016

Characteristic	Individuals, No. (%)			Normalized difference^a^
	All (April 2014 to December 2016)	Baseline (April 2014 to December 2014)	Enrollment assistance (January 2015 to December 2016)	
No. of releases^b^	18 265	4889	13 376	NA
No. of prisoners	16 307	4828	12 553	NA
Sex				
Female	1945 (10.6)	462 (9.4)	1483 (11.1)	0.054
Male	16 320 (89.4)	4427 (90.6)	11 893 (88.9)	
Age at release, mean (SD), y	35.5 (10.7)	35.1 (10.6)	35.6 (10.7)	0.047
Time incarcerated, mean (SD), mo	21.5 (29.2)	20.7 (27.9)	21.8 (29.6)	0.037
Race and ethnicity				
Black	6213 (34.0)	1693 (34.6)	4520 (33.8)	−0.018
White	10 969 (60.1)	2912 (59.6)	8057 (60.2)	0.014
Other^c^	1083 (5.9)	284 (5.8)	799 (6.0)	0.007
Educational level				
<High school or GED	4958 (27.1)	1404 (28.7)	3554 (26.6)	−0.048
≥High school or GED	12 465 (68.2)	3239 (66.3)	9226 (69.0)	0.058
Missing	842 (4.6)	246 (5.0)	596 (4.5)	−0.027
Marital status				
Single	15 921 (87.2)	4295 (87.9)	11 626 (86.9)	−0.028
Married	1757 (9.6)	444 (9.1)	1313 (9.8)	0.025
Other	587 (3.2)	150 (3.1)	437 (3.3)	0.11
Rurality of county of conviction				
Part of metropolitan statistical area	14 638 (80.1)	3918 (80.1)	10 720 (80.1)	0.000
Not part of metropolitan statistical area	3547 (19.4)	941 (19.2)	2606 (19.5)	0.006
Missing	80 (0.4)	30 (0.6)	50 (0.4)	−0.034
Type of release				
Supervision	16 499 (90.3)	4346 (88.9)	12 153 (90.9)	0.065
No supervision	616 (3.4)	159 (3.3)	457 (3.4)	0.009
Other	1150 (6.3)	384 (7.9)	766 (5.7)	−0.085
Release facility security status				
Minimum	6768 (37.1)	1711 (35.0)	5057 (37.8)	0.058
Medium	9113 (49.9)	2547 (52.1)	6566 (49.1)	−0.06
Medium and maximum	763 (4.2)	191 (3.9)	572 (4.3)	0.019
Maximum	1544 (8.5)	432 (8.8)	1112 (8.3)	−0.019
Jail	77 (0.4)	8 (0.2)	69 (0.5)	0.061
Paralegal benefits specialist at facility	5050 (27.6)	0	5050 (37.8)	NA
History of SUD or HCV				
Self-reported opioid use	2405 (13.2)	624 (12.8)	1781 (13.3)	0.016
At risk for or history of HCV	10 672 (58.4)	2701 (55.2)	7971 (59.6)	0.088
Highly probable need for SUD treatment	14 509 (80.2)	3864 (80.1)	10 645 (80.2)	0.003

Abbreviations: GED, General Educational Development Certification; HCV, hepatitis C virus; NA, not applicable; SUD, substance use disorder.

^a^ A normalized balance test compared the baseline period with the enrollment assistance period. It is calculated as the difference between mean values from each sample period scaled by a measure of their SD. No normalized difference was calculated for the paralegal benefits specialists because they were not present during the baseline period in any facility.

^b^ Percentages are calculated using the number of releases as the denominator.

^c^ Other races and ethnicities include American Indian or Alaska Native, Asian or Pacific Islander, and unknown.

Trends in the unadjusted outcomes show no indication of sudden downward shifts before implementation of enrollment assistance and no unusual patterns to suggest an association with confounding events (eFigure 3 in the Supplement). When we observed abrupt changes in the outcome trends, they typically coincided with implementation of enrollment assistance.

Table 2 compares the unadjusted percentages of adults released before and after implementation of enrollment assistance who received health care within 30 days of release. We observed statistically significant increases in all outcomes except ED visits. Use of any outpatient visit within 30 days was the most prevalent; the percentage of adults with any outpatient visit within 30 days of release increased from 16.1% (95% CI, 15.1%-17.1%) at baseline to 24.4% (95% CI, 23.6%-25.1%; *P* < .001) after the program’s implementation.

**Table 2.** Unadjusted Percentage of Adults Released From Prison With Any Health Care Use Within 30 Days After Incarceration (18 265 Releases)^a^

Study outcome	% (95% CI)		*P* value
	Baseline period	Enrollment assistance period	
Outpatient visit			
Any	16.1 (15.1 to 17.1)	24.4 (23.6 to 25.1)	<.001
With OUD diagnosis	0.7 (0.5 to 1.0)	1.4 (1.2 to 1.6)	<.001
With SUD diagnosis	2.5 (2.1 to 3.0)	3.8 (3.4 to 4.1)	<.001
Medication treatment for OUD	0.3 (0.1 to 0.4)	0.7 (0.5 to 0.8)	<.001
ED visit	5.6 (4.9 to 6.2)	6.2 (5.8 to 6.6)	.06
ED visit for overdose	0.3 (0.1 to 0.4)	0.4 (0.3 to 0.5)	.13
Inpatient stay	0.8 (0.6 to 1.1)	1.1 (0.9 to 1.3)	.04
Inpatient stay for overdose	0.06 (−0.008 to 0.13)	0.2 (0.1 to 0.2)	.048

Abbreviations: ED, emergency department; OUD, opioid use disorder; SUD, substance use disorder.

^a^ Authors’ calculations from Wisconsin Medicaid and Department of Corrections data. The unadjusted percentage of releases with each outcome is shown for the baseline and postenrollment assistance period. The *P* value for the test of equivalence across time periods is shown.

**Figure 1.**
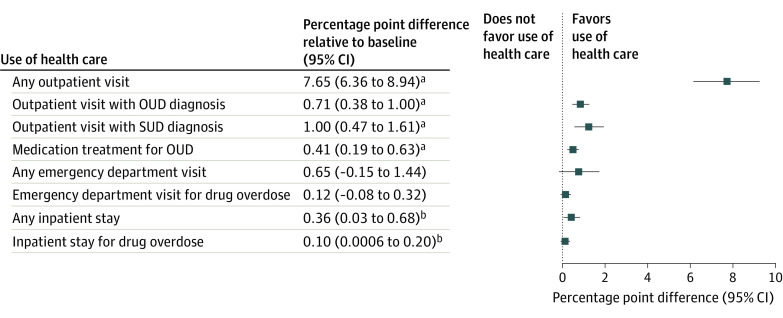
Association of the Availability of a Medicaid Enrollment Assistance Program With Use of Health Care Within 30 Days of Release From Prison: Results From Regression Analyses Authors’ calculations from Wisconsin Medicaid and Department of Corrections data. Each row in the table presents the results of a separate regression for the identified outcome, the point estimate, and its 95% CI. The adjacent graph plots the point estimate and its 95% CI relative to the dashed line at 0, which represents the null hypothesis (which is no change in health care use associated with being released after implementation of the enrollment assistance program relative to being released before its implementation). OUD indicates opioid use disorder; SUD, substance use disorder. ^a^*P* < .001. ^b^*P* < .05.

Figure 1 presents the absolute percentage point differences from the regression analyses in the expected outcome after implementation of enrollment assistance compared with the expected value of the outcome before its implementation. After implementation of enrollment assistance, the likelihood of any outpatient visit increased by 7.7 percentage points (95% CI, 6.4-8.9 percentage points; *P* < .001), a relative change of 47.8% (ie, 7.7 of 16.1), resulting in 23.8% of individuals (7.7 + 16.1 percentage points) receiving this service within 30 days of release. Use of outpatient care for the treatment of SUDs increased after implementation of enrollment assistance; the likelihood of any visit with an OUD diagnosis increased by 0.7 percentage points (95% CI, 0.4-1.0 percentage points; *P* < .001), the likelihood of any visit with an SUD diagnosis increased by 1.0 percentage point (95% CI, 0.5-1.6 percentage points; *P* < .001), and the likelihood of receipt of medication for OUD increased by 0.4 percentage points (95% CI, 0.2-0.6 percentage points; *P* < .001). Compared with the baseline period, these absolute increases translate into relative increases of 100%, 40%, and 133%, respectively. The probability of any inpatient stay increased by 0.4 percentage points (95% CI, 0.03-0.7 percentage points; *P* = .03), and the probability of an inpatient stay associated with drug overdose increased by 0.1 percentage point (95% CI, 0.0006-0.20 percentage points; *P* < .05). There was no significant change in the use of the ED (0.7 percentage points [95% CI, –0.02 to 1.4 percentage points]). Complete results are included in eTable 2 of the Supplement.

**Figure 2.**
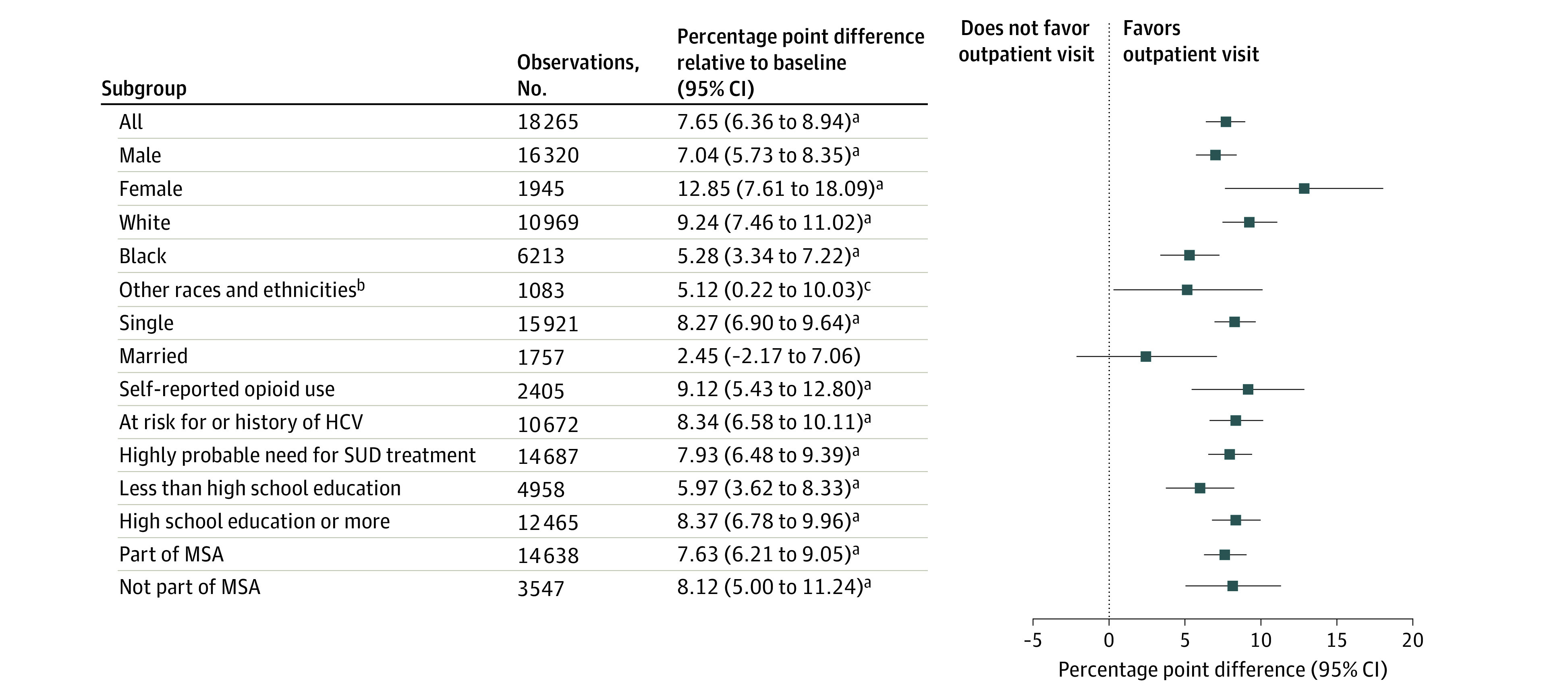
Association of the Availability of a Medicaid Enrollment Assistance Program With Any Outpatient Visit Within 30 Days of Release From Prison: Results From Regression Analyses by Subgroup Authors’ calculations from Wisconsin Medicaid and Department of Corrections data. Each row in the table presents the results of a separate regression for the identified subgroup, the point estimate, and its 95% CI. The adjacent graph plots the point estimate and its 95% CI relative to the dashed line at 0, which represents the null hypothesis (which is no change in health care use associated with being released after implementation of the enrollment assistance program relative to being released before its implementation). HCV indicates hepatitis C virus; MSA, metropolitan statistical area; and SUD, substance use disorder. ^a^*P* < .001. ^b^Other races and ethnicities include American Indian or Alaska Native, Asian or Pacific Islander, and unknown. ^c^*P* < .05.

The positive and statistically significant association between exposure to the Medicaid enrollment assistance program and the likelihood of any outpatient visit held for all subgroups except married adults (Figure 2). The magnitude of increases in the likelihood of any outpatient visit relative to the baseline mean ranged from 5.1 percentage points (95% CI, 0.22-10.0 percentage points; *P* = .04) among adults of other races to 12.8 percentage points (95% CI, 7.6-18.1 percentage points; *P* < .001) among women.

Results from the sensitivity analyses restricted to first releases and models that included facility fixed effects were consistent with the main findings (eTables 4-6 in the Supplement). Results were also consistent across programs with or without a part-time benefits specialist (eTable 3 in the Supplement).

## Discussion

We examined the association between a newly available prerelease Medicaid enrollment assistance program for adults incarcerated in state prison and health care use during the immediate postrelease period among those with a history of substance use. There are 3 key findings. First, the presence of a prison-based Medicaid enrollment assistance program was associated with large absolute increases in the likelihood of having any outpatient visit within 30 days after release. Second, despite large relative increases for outpatient care associated with SUDs, the absolute levels of this health care use in the immediate postincarceration period remained low. Third, there was no evidence of accompanying reductions in the use of hospital-based care.

After implementation of prerelease Medicaid enrollment assistance, the likelihood of having any outpatient visit during the first 30 days after incarceration among adults with a history of substance use increased by 7.7 percentage points, a relative change of 47.8%. Large positive increases were consistent across subgroups (Figure 2). In total, approximately 24% of the population had an outpatient visit within 30 days of release after the enrollment assistance program was implemented. Different stakeholders may interpret this value as higher or lower than expected. To our knowledge, only 1 study has used administrative data to assess this outcome; 44% of adults released from an Australian prison had an outpatient visit within 30 days.^43^ The generalizability of this finding to a US context is unclear.

The absolute rates of postincarceration health care use associated with substance use are low, given the population’s history of substance use. After implementation of enrollment assistance, 3.8% of individuals had an SUD-associated outpatient visit within 30 days of release (Table 2). Although increasing Medicaid enrollment is an important step in ensuring needed treatment, further interventions may be required to address other barriers to accessing care. These barriers include limited health literacy,^44^ competing social and economic priorities that formerly incarcerated adults face,^45^ and logistical challenges^46^ that may not be equally distributed across all adults leaving prison. A network of transitional care clinics is an important resource for formerly incarcerated adults, although connecting individuals leaving prison with these clinics remains a challenge.^47^ Transitional care interventions, including case management and peer navigation, offer promising strategies^48,49,50,51,52^ that warrant investigation regarding how to bring them to scale.

A prompt connection to treatment on release from prison may be lifesaving for adults with SUDs by preventing a drug overdose. Although we observed increased use of outpatient health care that may facilitate this treatment, we found no evidence of decreased use of hospital-based services. The direction of results for ED and inpatient stays was positive and, for inpatient stays, was statistically significant though very small in absolute magnitude (Figure 1). We consider several possible explanations. Individuals who are at highest risk of a drug overdose may be relatively less likely to obtain outpatient care without support or intervention that goes beyond health insurance. We observed variation in the relative increases in outpatient care use by sample subgroup (Figure 2), which provides a starting point for further inquiry. Alternatively, the type or timing of outpatient health care received may have been insufficient to mitigate acute events. Future research that characterizes the health and social characteristics of individuals who do or do not seek postrelease care and the nature of the care received will be important to crafting care transition interventions that reduce the risk of drug overdose.

### Limitations

This study has several limitations. First, in this observational cohort study, we cannot rule out the possibility of confounding due to contemporaneous events. Second, we observed only Medicaid-reimbursed health care; results may thus overstate the association between the enrollment assistance program and total health care use if individuals obtained care paid by other sources (eg, private insurance or charity care). Although we cannot rule out the possibility, we think this scenario is unlikely given the persistent barriers to care among uninsured adults even within publicly funded health centers^53,54,55^ and the low rate of adults released from state prison who have private insurance coverage within 2 to 3 months of release (ie, 4%-6%).^5^ Third, our definition of a history of substance use has not been clinically validated. However, the prevalence of a history of substance use in our sample is consistent with estimates from other prison populations, suggesting reasonable face validity.^4^ Fourth, the generalizability of our findings may be limited by the attributes of this state’s Medicaid program, the enrollment assistance program, and the short duration of follow-up. Fifth, claims-based outcomes do not convey the nature of the encounter or whether it adequately addresses the individual’s health need.

## Conclusions

This cohort study’s findings reinforce the importance of implementing the Medicaid reentry provision in the 2018 SUPPORT Act,^11^ which supports state efforts to provide prerelease Medicaid enrollment assistance in prisons and jails. Currently, adults leaving correctional facilities face different Medicaid enrollment opportunities depending on the Medicaid expansion status of their state and the availability and type of enrollment assistance at their correctional facility.^56^ Although there remains substantial room for improvement in access to SUD-specific care, our results suggest that such assistance translates into improved access to outpatient health care after incarceration, consistent with the policy aims of the SUPPORT Act.

## Notes

Supplement. **eTable 1.** Sample Construction **eAppendix 1.** Enrollment Assistance Program **eFigure 1.** Medicaid Enrollment in the Month of Release for Adults With a History of Substance Use, April 2014-December 2016 **eAppendix 2.** History of Substance Use Definition **eFigure 2.** Prevalence of Indicators of History of Substance Use in the Analytical Sample **eAppendix 3.** Health Care Use Measures **eFigure 3.** Trends in Unadjusted Outcomes **eAppendix 4.** Empirical Model Specification **eTable 2.** Complete Intent to Treat Regression Results **eTable 3.** Predicted Change in Postrelease Care Use With and Without Benefits Specialist **eTable 4.** ITT Regression Results Restricted to First Release per Subject **eTable 5.** Unadjusted Outcomes Restricted to First Release per Subject, N=16,307 Releases **eTable 6.** ITT Regression Results With Facility Fixed Effects
eReferences.
