# Supplementary material for: Association Between Assistance With Medicaid Enrollment and Use of Health Care After Incarceration Among Adults With a History of Substance Use
Source: JAMA Netw Open. 2022 Jan 7;5(1):e2142688. doi: 10.1001/jamanetworkopen.2021.42688 (PMC8742194; doi:10.1001/jamanetworkopen.2021.42688)
Supplement: Supplement. — eTable 1. Sample Construction eAppendix 1. Enrollment Assistance Program eFigure 1. Medicaid Enrollment in the Month of Release for Adults With a History of Substance Use, April 2014-December 2016 eAppendix 2. History of Substance Use Definition eFigure 2. Prevalence of Indicators of History of Substance Use in the Analytical Sample eAppendix 3. Health Care Use Measures eFigure 3. Trends in Unadjusted Outcomes eAppendix 4. Empirical Model Specification eTable 2. Complete Intent to Treat Regression Results eTable 3. Predicted Change in Postrelease Care Use With and Without Benefits Specialist eTable 4. ITT Regression Results Restricted to First Release per Subject eTable 5. Unadjusted Outcomes Restricted to First Release per Subject, N=16,307 Releases eTable 6. ITT Regression Results With Facility Fixed Effects eReferences. [file jamanetwopen-e2142688-s001.pdf]

Supplementary Online Content

Burns ME, Cook S, Brown LM, et al. Association between assistance with Medicaid enrollment and use of health care after incarceration among adults with a history of substance use. *JAMA Netw Open.* 2022;5(1):e2142688. doi:10.1001/jamanetworkopen.2021.42688

eTable 1. Sample Construction

eAppendix 1. Enrollment Assistance Program

eFigure 1. Medicaid Enrollment in the Month of Release for Adults With a History of Substance Use, April 2014-December 2016

eAppendix 2. History of Substance Use Definition

eFigure 2. Prevalence of Indicators of History of Substance Use in the Analytical Sample

eAppendix 3. Health Care Use Measures

eFigure 3. Trends in Unadjusted Outcomes

eAppendix 4. Empirical Model Specification

eTable 2. Complete Intent to Treat Regression Results

eTable 3. Predicted Change in Postrelease Care Use With and Without Benefits Specialist

eTable 4. ITT Regression Results Restricted to First Release per Subject

eTable 5. Unadjusted Outcomes Restricted to First Release per Subject, N=16,307 Releases

eTable 6. ITT Regression Results With Facility Fixed Effects

eReferences.

This supplementary material has been provided by the authors to give readers additional information about their work.

eTable 1. Sample Construction

eTable1 describes the construction of the study sample beginning with all releases from Wisconsin state correctional facilities between 4/1/2014 – 6/30/2017 after an incarceration duration of greater than or equal to 31 days.

**Inclusion/Exclusion criteria**	**N prison releases**	**N persons**
Released 04/01/2014 - 12/31/2016	25486	22874
Released to WI community	25360	22751
Non-missing facility id	25358	22750
Non-missing facility security level	25305	22698
History of substance use	18265	16307

© 2022 Burns ME et al. *JAMA Network Open.*

eAppendix 1. Enrollment Assistance Program

Beginning in January 2015, the WI Department of Corrections (DOC) implemented pre-release Medicaid enrollment assistance. The program served adults under the supervision of the state's Division of Adult Institutions (DAI) incarcerated within state correctional facilities; these include state prisons, correctional centers, and DAI-contracted beds within county jails. Under the enrollment assistance program, individuals may apply for Medicaid as early as the 20th day of the month prior to their month of release which allows time for the Wisconsin Department of Health Services to send the individual's Medicaid card to their institution before release.

In all facilities, discharge planning staff provide guidance to individuals on how to apply for Medicaid, and individuals are then given the opportunity to call an eligibility case worker from the correctional facility to do so. Additionally, five facilities share three paralegal benefit specialists to assist with the enrollment process. The DOC selected these five facilities based on the composition of their populations (e.g., relatively high prevalence of limited English proficiency, intellectual disabilities, mental illness, etc.)

The off-site eligibility caseworkers who field inmates' calls are employed by regional Income Maintenance Agencies. Eligibility is typically determined during the initial call. The caseworker verifies information provided by the applicant using information exchanges, collects an electronic signature, determines eligibility, and notifies the applicant of the outcome. If deemed eligible, the Medicaid coverage is effective upon release.

As previously reported, following the program's implementation, Medicaid enrollment in the month of release increased by 25 percentage points.[1] This increase was driven by an abrupt uptick in enrollment applications that were dated before the release date. The timing of applications followed by confirmed Medicaid enrollment in the month of release, strongly suggests that Medicaid coverage was operative *upon* release because eligibility decisions were generally reached on the date of application.

The Medicaid enrollment status for this study's sample across the study periods is shown below in eFigure 1.

© 2022 Burns ME et al. *JAMA Network Open.*

eFigure 1. Medicaid Enrollment in the Month of Release for Adults With a History of Substance Use, April 2014-December 2016

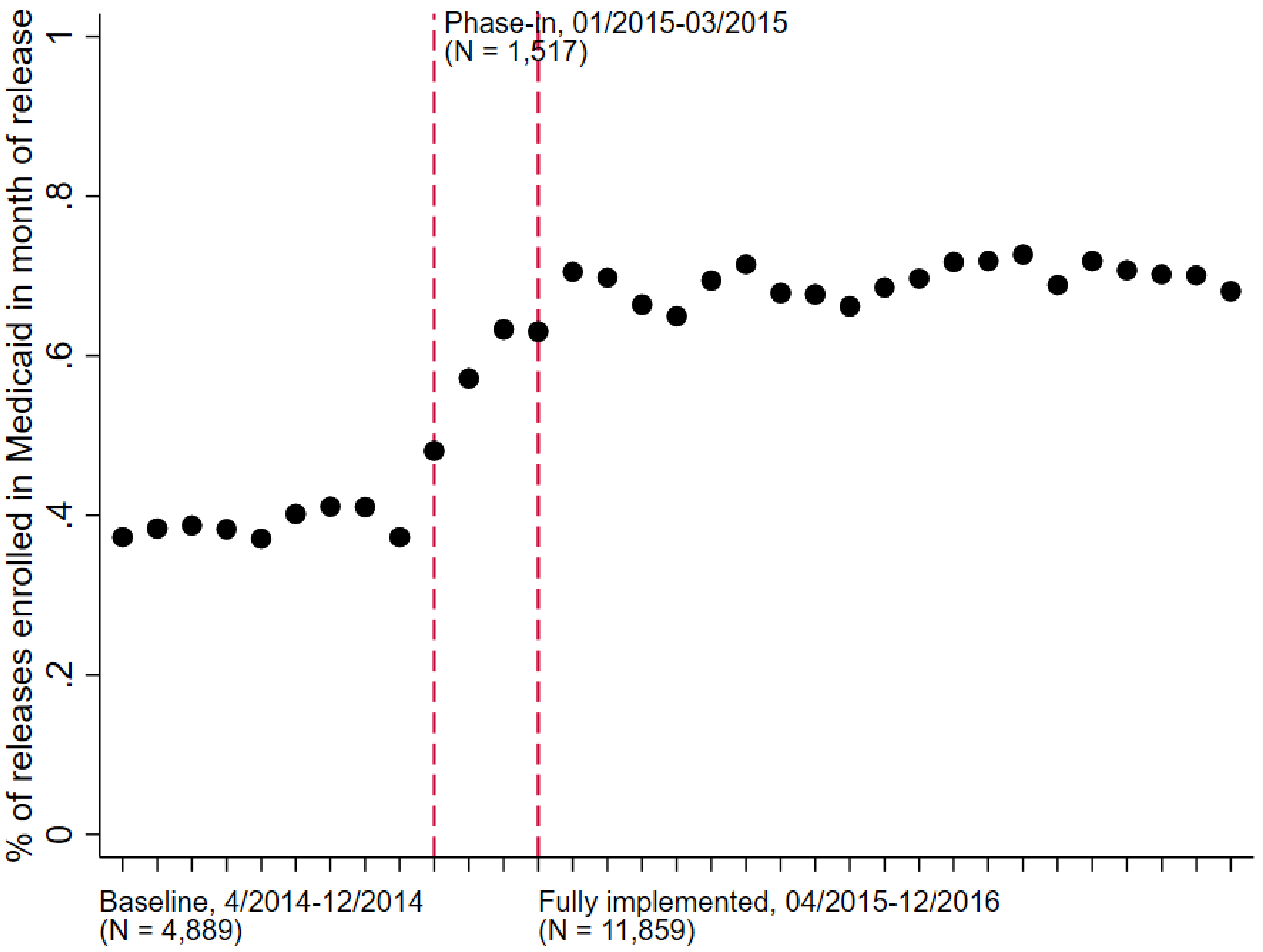

Note: Black dotes show percent of individuals released in the corresponding month who enrolled in Medicaid within one month of release. First dashed red line marks the beginning of the phase-in of the enrollment assistance program. Second dashed red line marks the beginning of full implementation of the enrollment assistance program.

© 2022 Burns ME et al. *JAMA Network Open.*

eAppendix 2. History of Substance Use Definition

Identifying "highly probable" need for substance use treatment

The underlying function of the COMPAS instrument is to assess risk of recidivism including potentially modifiable correlates of recidivism including substance use.[2,3] Available assessments of the validity of the COMPAS substance use score concern the degree to which this score is associated with recidivism rather than a clinical diagnosis of substance use disorder.[2]

During our study period, the Wisconsin Department of Corrections (WI DOC) was adopting the COMPAS with the eventual goal of collecting two COMPAS assessments per person: one using the COMPAS Core instrument at intake; and one using the COMPAS Reentry instrument close to the time of release. During this implementation process, it was frequently the case that individuals completed just one assessment – either Core or Reentry – depending on the time of administration. Thus, for each subject we obtained from the WI DOC the most recently completed COMPAS assessment relative to the individual's release date, and no more than 120 days after their release. An assessment may have a date after the release if it was conducted through the community supervision program.

There are some differences in the Core and Reentry instruments with respect to the substance use history questions although the WI DOC generates the same 3-category score indicating a need for treatment from each instrument: highly probable, probable, and unlikely. The specific questions on which this score is based for each instrument are noted below. We do not have access to the proprietary algorithm used to generate the score. However, in our internal analysis the vast majority of individuals identified as "highly probable" using the Core instrument had three or more positive responses to the substance use history questions. Using the Reentry instrument, the vast majority of individuals identified as highly probable had five or more positive response to the substance use history questions.

<u>CORE Instrument Substance Use History Questions</u>

1.Do you think your current/past legal problems are partly because of alcohol or drugs?
2.Were you using alcohol when arrested for your current offense?
3.Were you using drugs when arrested for your current offense?
4.Are you currently in formal treatment for alcohol or drugs such as counseling, outpatient, inpatient, residential?
5.Have you ever been in formal treatment for alcohol such as counseling, outpatient, inpatient, residential?
6.Have you ever been in formal treatment for drugs such as counseling, outpatient, inpatient, residential?
7.Do you think you would benefit from getting treatment for alcohol?
8.Do you think you would benefit from getting treatment for drugs?
9.Did you use heroin, cocaine, crack or methamphetamines as a juvenile?

<u>COMPAS Reentry Instrument Substance Use History Questions</u>

1.Committed Offenses while high/drunk?
2.Prior drug charges/convictions?
3.History of drug problems?
4.History of alcohol problems?
5.Prior treatments for drug/alcohol abuse?
6.Any history of failed drug/urine analysis test?
7. Is the inmate at risk for substance abuse problems?

Identifying individuals with self-reported opioid use

The COMPAS Substance Abuse Module, which is not used in the above algorithm, asks individuals what substances they have a history of using. From the list of self-reported substances, the following are identified as opiates: buprenorphine, codeine, fentanyl, heroin, methadone, morphine, opiates, and Vicodin. Staff at the WI DOC, which include staff from WI DOC community corrections programs, are required to enter data into this assessment at intake. When an individual is reincarcerated or placed under community supervision, information may be added to this assessment. Thus, data on substances ever used likely became more complete over time (i.e. data are cumulative).

WI DOC staff members collecting the COMPAS data, record all substances the individual self-reports having used. Although the purpose of this data point is to understand problematic drug use (e.g., misuse of prescription drugs and illicit drug use), it is possible that respondents interpret the question differently and report opioids used for pain management. We believe this scenario is uncommon because DOC staff collecting the data are aware of the goal of the question. However, we cannot guarantee that people who used opioids strictly for pain management have been entirely excluded.

© 2022 Burns ME et al. *JAMA Network Open.*

Risk of Hepatitis C Virus as proxy for injection drug use.

We defined persons living with, or at risk of HCV, as those who received a prescription medication for HCV while incarcerated according to Corrections' pharmacy claims, received a referral to receive an HCV test upon prison admission according to the State Lab of Hygiene, or had ever had a positive antibody test for HCV before release from prison based on state surveillance data. Living with, or at risk of, HCV is an imperfect proxy for having a history of injection drug use. It is possible that individuals living with HCV were infected through other means of transmission (e.g., blood transfusion). The DOC's risk-based screening instrument that determines HCV testing upon intake may capture people born between 1945-1965 ("baby boomers"), who have no other behavioral risks (Stockman et al. 2016). However, because individuals who are incarcerated likely have a different risk profile for HCV than the general baby boomer population, we expect most individuals living with, or at risk of, HCV in this study to have a history of injection drug use.

© 2022 Burns ME et al. *JAMA Network Open.*

eFigure 2. Prevalence of Indicators of History of Substance Use in the Analytical Sample

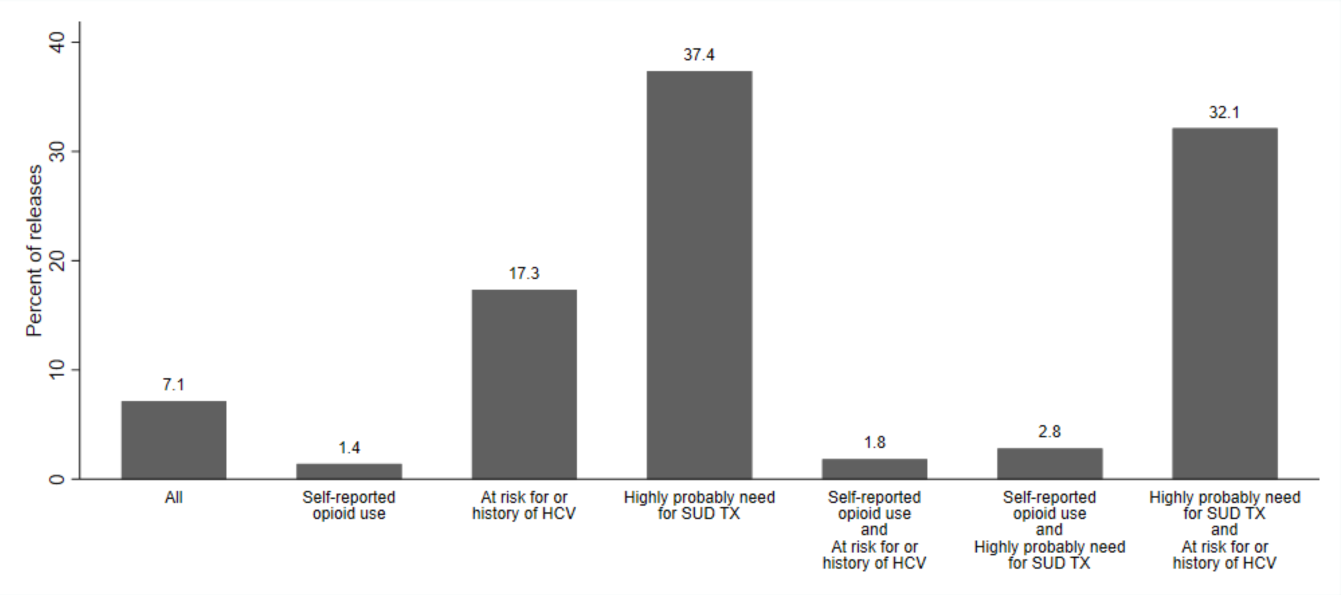

© 2022 Burns ME et al. *JAMA Network Open.*

eAppendix 3. Health Care Use Measures

We adopt the diagnosis and procedure codes published by the Medicaid Outcomes Distributed Research Network[4] to define visits for OUD and SUD, as well as medications for opioid use disorder. An outpatient visit is considered OUD- or SUD-related based on the presence of one of the relevant diagnoses shown below in any position on the claim.

<u>Opioid Use Disorder</u>

- ICD-9: 304.0x, 305.5x
- ICD-10: F11.xxx

<u>Substance Use Disorders</u>

- ICD-9: 303-305, exclude Tobacco 3051; exclude remission codes (5th digit = \`3’)
- ICD-10: F10-F19, exclude Tobacco F17, exclude remission codes; F55, O355, o9931, O9932

<u>Medications for Opioid Use Disorder</u>

- A prescription claim for: buprenorphine, Naltrexone (oral), Injectable Naltrexone, or buprenorphine/Naloxone; *or*
- A HCPCS code for buprenorphine or buprenorphine/Naloxone, oral: J0571, J0573, J0574, J0575; methadone administration, H0020; Naltrexone (extended-release injectable): J2315.

© 2022 Burns ME et al. *JAMA Network Open.*

eFigure 3. Trends in Unadjusted Outcomes

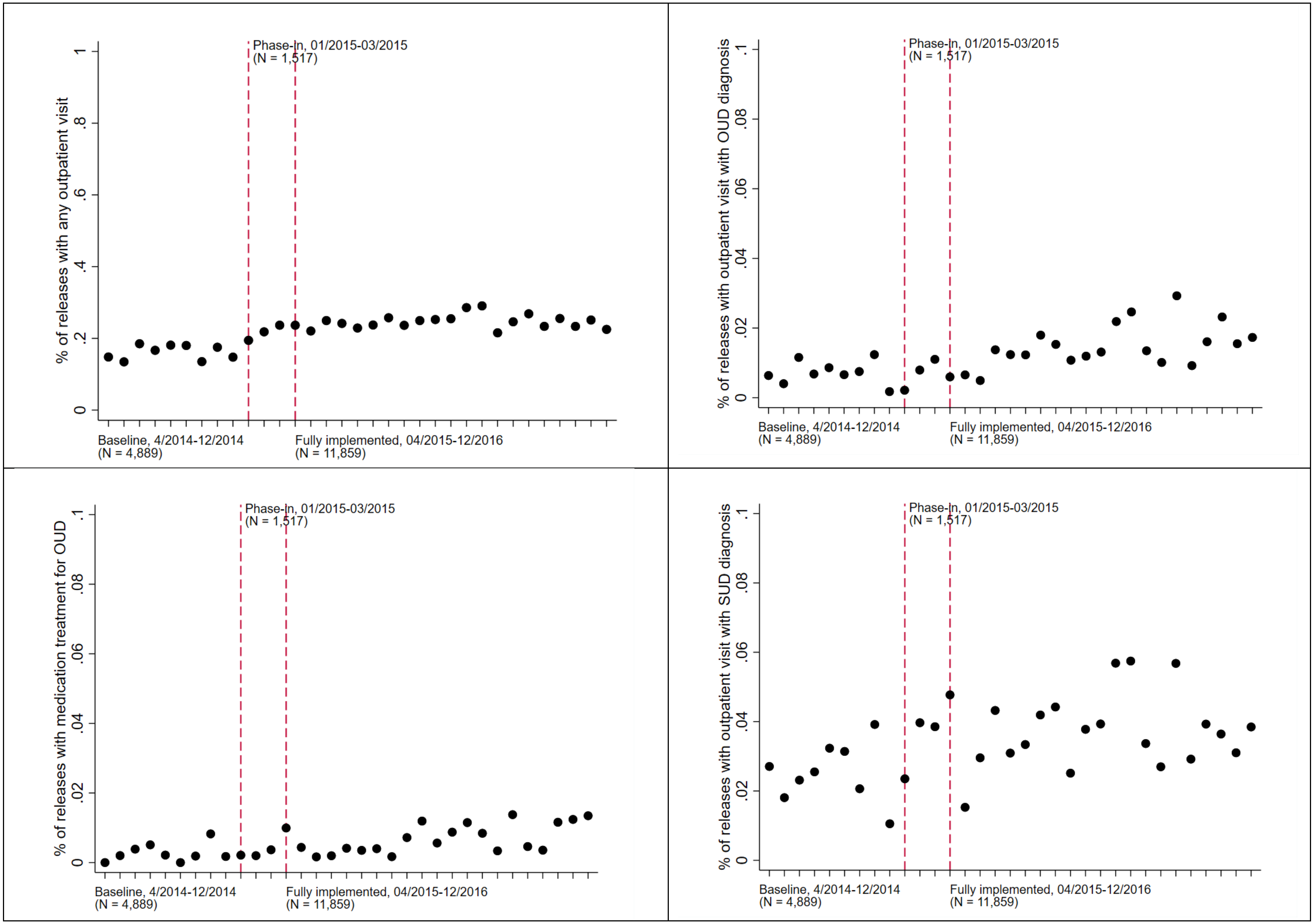

© 2022 Burns ME et al. *JAMA Network Open.*

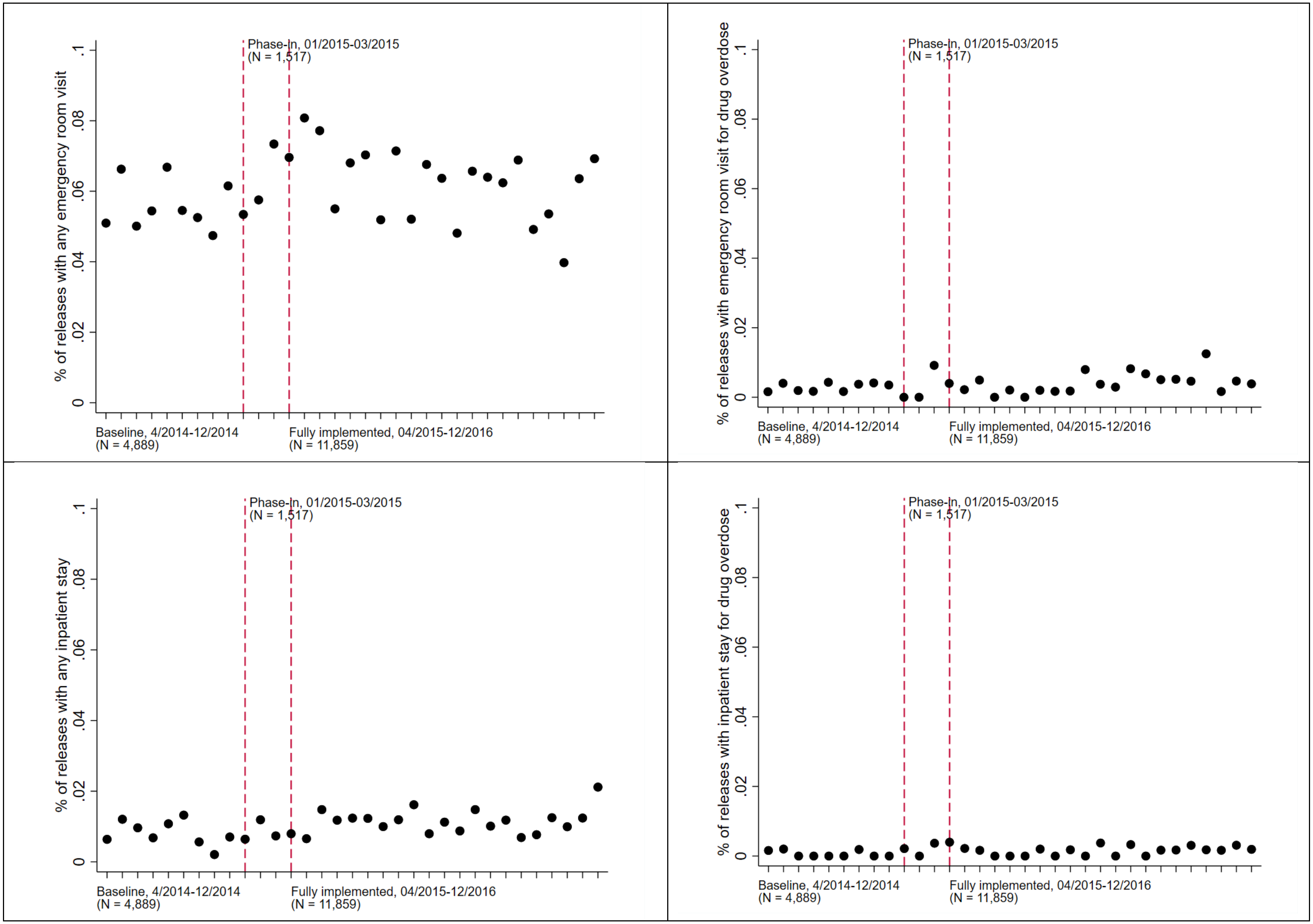

© 2022 Burns ME et al. *JAMA Network Open.*

eAppendix 4. Empirical Model Specification

Our main regression model takes the following general form:

$$Y_{it} = \alpha_0 + \alpha_1 PHpara + \alpha_2 PHnopara + \alpha_3 Fullimp + X_{it}\theta + P_{it}\sigma + \varepsilon_{it}$$

Y is the outcome, i indexes a release from prison in month-year t, adjusts for a vector of X_{it} individual characteristics, a vector of P_{it} control variables that are specific to the prison-release including duration of incarceration, and type of release, and ϵ_{it} represents a random error. Three policy variables characterize the enrollment assistance program; PHpara = 1 during the phase-in period (Jan 2015 – March 2015) for facilities in which a part-time benefits specialist is present; PHnopara =1 during the phase-in period (Jan 2015-March 2015 for facilities in which no part-time benefit specialist is present; and Fullimp =1 for all facilities during the after the program is fully implemented (>=April 2015). The coefficient of interest is α_3. It reflects the average change in the outcome after implementation of enrollment assistance compared to the baseline period.

To obtain separate predictions for fully implemented enrollment assistance programs with and without the inclusion of a part-time benefits specialist, we modified the specification to take the following form:

$$Y_{it} = \alpha_0 + \alpha_1 PHpara + \alpha_2 PHnopara + \alpha_3 Fullimppara + \alpha_4 Fullimpnopara + X_{it}\theta + P_{it}\sigma + \varepsilon_{it}$$

Y is the outcome, i indexes a release from prison in month-year t, adjusts for a vector of X_{it} individual characteristics, a vector of P_{it} control variables that are specific to the prison-release including duration of incarceration, and type of release, and ϵ_{it} represents a random error. Three policy variables characterize the enrollment assistance program; PHpara = 1 during the phase-in period (Jan 2015 – March 2015) for facilities in which a benefits specialist is present; PHnopara =1 during the phase-in period (Jan 2015-March 2015 for facilities in which no benefit specialist is present; and Fullimppara =1 during the full implementation period (>=April 2015) for all facilities in which a part-time benefits specialist was present; Fullimpnopara=1 during the full implementation period (>=2015) for all facilities in which no part-time benefits specialist was present.

© 2022 Burns ME et al. *JAMA Network Open.*

eTable 2. Complete Intent to Treat Regression Results

	(1)	(2)	(3)	(4)	(5)	(6)	(7)	(8)
	**Any outpatient visit**	**Outpatient visit with OUD diagnosis**	**Outpatient visit with SUD diagnosis**	**Medication treatment for OUD**	**ED visit**	**ED visit for overdose**	**Inpatient stay**	**Inpatient stay for overdose**
Phase-in, no benefits specialist	0.0161	-0.000207	-0.00233	-0.000290	0.00923	-0.00115	0.00290	0.00121
	(0.0174)	(0.00413)	(0.00788)	(0.00289)	(0.0103)	(0.00246)	(0.00408)	(0.00147)
Phase-in, benefits specialist	0.0457*	-0.00375	0.00378	-0.00451	0.00798	0.00159	0.00130	0.00302
	(0.0219)	(0.00585)	(0.0106)	(0.00357)	(0.0130)	(0.00374)	(0.00489)	(0.00271)
Full implementation	0.0765**	0.00706**	0.0104**	0.00407**	0.00645	0.00120	0.00355*	0.00103*
	(0.00658)	(0.00170)	(0.00291)	(0.00113)	(0.00405)	(0.00100)	(0.00166)	(0.000520)
Female	0.198**	0.0336**	0.0397**	0.0122**	0.0370**	-0.00224	0.00517	-0.000502
	(0.0150)	(0.00600)	(0.00789)	(0.00356)	(0.00827)	(0.00165)	(0.00339)	(0.000994)
Age	0.00567**	-0.000338**	0.000309*	-0.0000766	0.00143**	0.0000232	0.000439**	-0.00000593
	(0.000309)	(0.0000591)	(0.000122)	(0.0000471)	(0.000186)	(0.0000422)	(0.0000836)	(0.0000220)
Black	-0.0823**	-0.0148**	-0.0269**	-0.00596**	0.00295	-0.00440**	-0.00611**	-0.000926
	(0.00666)	(0.00148)	(0.00253)	(0.00106)	(0.00420)	(0.000964)	(0.00172)	(0.000525)
Other	-0.0724**	-0.0135**	-0.00916	-0.00627**	-0.000677	-0.00560**	-0.00571*	-0.00206**
	(0.0127)	(0.00285)	(0.00634)	(0.00190)	(0.00791)	(0.000767)	(0.00264)	(0.000515)
>= H.S.	0.00210	0.00266	0.00674*	0.000849	-0.0101*	-0.00259*	-0.00111	-0.000244
	(0.00687)	(0.00167)	(0.00281)	(0.00117)	(0.00433)	(0.00118)	(0.00173)	(0.000611)
Missing Education	0.0220	0.00640	0.00799	0.00445	-0.0166	-0.00562**	-0.00333	-0.00183
	(0.0180)	(0.00562)	(0.00848)	(0.00405)	(0.00973)	(0.00185)	(0.00398)	(0.00186)
Married	-0.0140	-0.0000504	0.00147	0.0000149	-0.00595	-0.000521	0.00166	0.00184
	(0.0109)	(0.00261)	(0.00484)	(0.00180)	(0.00612)	(0.00145)	(0.00293)	(0.00133)
Other	0.00626	0.00268	0.00781	0.00417	0.0280*	0.00289	0.00963	0.00536
	(0.0206)	(0.00640)	(0.00970)	(0.00500)	(0.0127)	(0.00321)	(0.00597)	(0.00344)
Not part of MSA	0.0272**	-0.00262	0.00944*	-0.000191	-0.00634	0.0000448	-0.00147	0.00105
	(0.00845)	(0.00232)	(0.00411)	(0.00160)	(0.00469)	(0.00133)	(0.00197)	(0.000871)
Missing MSA	-0.108**	0.0188	0.0268	0.00133	-0.0256	0.000265	-0.00353	0.000494
	(0.0415)	(0.0136)	(0.0221)	(0.00221)	(0.0258)	(0.00105)	(0.0111)	(0.000594)
Months incarcerated	0.000178	-0.0000691**	-0.0000977*	-0.0000296	0.0000179	-0.0000320**	0.0000259	-0.0000101
	(0.000114)	(0.0000171)	(0.0000428)	(0.0000173)	(0.0000648)	(0.00000958)	(0.0000338)	(0.00000530)
No Supervision	-0.0552**	-0.00825**	-0.0226**	-0.00394*	0.00740	0.00156	0.0110	0.00200
	(0.0152)	(0.00192)	(0.00406)	(0.00176)	(0.0108)	(0.00288)	(0.00611)	(0.00234)
Other	-0.0209	-0.00312	-0.0141**	-0.00375	-0.00635	0.000688	0.00471	0.000123
	(0.0120)	(0.00325)	(0.00424)	(0.00193)	(0.00768)	(0.00184)	(0.00361)	(0.00127)
Medium	0.0422**	0.00403**	-0.000719	0.00405**	0.0211**	-0.000510	0.00587**	0.000410
	(0.00695)	(0.00155)	(0.00296)	(0.00105)	(0.00384)	(0.000961)	(0.00163)	(0.000595)
Med/max	0.137**	0.0171	0.0474**	0.0117	0.0589**	0.00669	0.00910	0.00317
	(0.0227)	(0.0105)	(0.0143)	(0.00681)	(0.0146)	(0.00380)	(0.00576)	(0.00240)

© 2022 Burns ME et al. *JAMA Network Open.*

Maximum	0.0366**	0.000363	-0.00746	0.00357*	0.0569**	-0.000270	0.0187**	-0.000273
	(0.0111)	(0.00215)	(0.00428)	(0.00182)	(0.00806)	(0.00174)	(0.00401)	(0.000759)
Jail	-0.0483	-0.0105**	-0.0353**	-0.00334**	-0.0233	0.00835	0.0217	0.0118
	(0.0375)	(0.00174)	(0.00334)	(0.000900)	(0.0135)	(0.0129)	(0.0181)	(0.0129)
Constant	-0.0562**	0.0159**	0.0157	0.00701	-0.0132	0.00721*	-0.0155**	-0.000295
	(0.0208)	(0.00495)	(0.00889)	(0.00427)	(0.0120)	(0.00332)	(0.00498)	(0.00147)
Observations	18265	18265	18265	18265	18265	18265	18265	18265
R^2	0.076	0.023	0.025	0.009	0.013	0.003	0.007	0.002

*Notes: The coefficients shown reflect the absolute difference in the outcome compared to the reference category for categorical and binary variables. For continuous measures, the coefficient reflects the absolute difference in the outcome for a one-unit increase. The first three rows describe policy variables that characterize the enrollment assistance program: the phase-in period (Jan 2015 – March 2015) with and without a benefit specialist, and the fully implemented period that doesn't differentiate between programs with and without a benefits specialist (>= April 2015). OUD indicates opioid use disorder, SUD indicates substance use disorder, and ED indicates emergency room. For example, relative to the baseline period, the likelihood of any outpatient visit increased by 7.65 percentage points (.0765 * 100) for person-releases exposed to the fully implemented enrollment assistance program. Standard errors in parentheses.* * $p < 0.05$, ** $p < 0.01$.

© 2022 Burns ME et al. *JAMA Network Open.*

eTable 3. Predicted Change in Postrelease Care Use With and Without Benefits Specialist

**Outcome**	**Difference between ITT-predicted outcome estimate and predicted baseline mean, Percentage Points (95% CI)**		**P-Value for Test of Equivalence between Columns A & B**
	**(A) Enrollment Assistance Program staffed by facility personnel & part-time benefit specialist**	**(B) Enrollment Assistance Program staffed by facility personnel**	
Any outpatient visit	8.73 (6.94, 10.51)	6.97 (5.53, 8.41)	0.067
Outpatient visit with OUD diagnosis	1.32 (0.76, 1.87)	0.32 (0.003, .65)	<.001
Outpatient visit with SUD diagnosis	1.82 (0.99, 2.64)	0.56 (-0.05, 1.17)	0.003
Medication treatment for OUD	0.64 (0.26, 1.02)	0.26 (0.03, .49)	0.070
Any emergency department visit	.93 (-0.18, 2.03)	0.47 (-0.39, 1.33)	0.421
Emergency department visit for drug overdose	0.36 (0.07, 65)	-0.03 (-0.24, 0.18)	0.007
Any inpatient stay	0.48 (0.03, 0.93)	0.28 (-0.08, 0.64)	0.398
Inpatient stay for drug overdose	0.07 (-0.08, 0.22))	0.12 (-0.001, 0.24)	0.604

Authors' calculations from Wisconsin Medicaid and Department of Corrections data. The differences shown are estimated from the main regression model for each outcome. The results may be interpreted as the absolute difference between the ITT predicted estimate and the predicted baseline value. OUD indicates opioid use disorder, and SUD indicates substance use disorder.

© 2022 Burns ME et al. *JAMA Network Open.*

eTable 4. ITT Regression Results Restricted to First Release per Subject

	(1)	(2)	(3)	(4)	(5)	(6)	(7)	(8)
	**Any outpatient visit**	**Outpatient visit with OUD diagnosis**	**Outpatient visit with SUD diagnosis**	**Medication treatment for OUD**	**ED visit**	**ED visit for overdose**	**Inpatient stay**	**Inpatient stay for overdose**
Phase-in, no benefits specialist	0.0155	-0.000923	0.000483	0.000384	0.00691	-0.00154	0.00320	0.000563
	(0.0180)	(0.00431)	(0.00815)	(0.00293)	(0.0104)	(0.00241)	(0.00415)	(0.00158)
Phase-in, benefits specialist	0.0433	-0.00413	0.00618	-0.00397	0.00887	0.00179	0.00219	0.00261
	(0.0229)	(0.00631)	(0.0112)	(0.00372)	(0.0136)	(0.00391)	(0.00511)	(0.00293)
Full implementation	0.0733**	0.00659**	0.00927**	0.00364**	0.00337	-0.000412	0.00210	0.000648
	(0.00688)	(0.00180)	(0.00307)	(0.00118)	(0.00420)	(0.000953)	(0.00172)	(0.000497)
Female	0.203**	0.0338**	0.0399**	0.0142**	0.0398**	-0.00290*	0.00402	-0.000243
	(0.0155)	(0.00618)	(0.00818)	(0.00386)	(0.00850)	(0.00119)	(0.00331)	(0.00106)
Age	0.00549**	-0.000295**	0.000305*	-0.0000686	0.00137**	0.0000173	0.000427**	-0.00000715
	(0.000316)	(0.0000611)	(0.000127)	(0.0000496)	(0.000191)	(0.0000386)	(0.0000856)	(0.0000188)
Black	-0.0811**	-0.0139**	-0.0257**	-0.00497**	0.00432	-0.00306**	-0.00616**	-0.000625
	(0.00686)	(0.00153)	(0.00265)	(0.00109)	(0.00432)	(0.000897)	(0.00167)	(0.000474)
Other	-0.0719**	-0.0128**	-0.00628	-0.00521*	0.00234	-0.00429**	-0.00408	-0.00181**
	(0.0129)	(0.00292)	(0.00676)	(0.00206)	(0.00798)	(0.000728)	(0.00289)	(0.000526)
>= H.S.	0.00139	0.00103	0.00534	-0.000247	-0.0111*	-0.00178	-0.000627	-0.000320
	(0.00711)	(0.00180)	(0.00298)	(0.00124)	(0.00450)	(0.00108)	(0.00174)	(0.000610)
Missing Education	0.0398*	0.00804	0.0151	0.00570	-0.0202*	-0.00311**	-0.00412	-0.00275
	(0.0202)	(0.00711)	(0.00991)	(0.00506)	(0.0100)	(0.00116)	(0.00384)	(0.00220)
Married	-0.0135	-0.000114	0.00325	0.000997	-0.00332	-0.000431	0.00385	0.00232
	(0.0111)	(0.00269)	(0.00505)	(0.00193)	(0.00636)	(0.00131)	(0.00315)	(0.00144)
Other	-0.0101	0.00278	0.00555	0.00484	0.0249	0.000833	0.00645	0.00530
	(0.0231)	(0.00809)	(0.0112)	(0.00625)	(0.0135)	(0.00176)	(0.00558)	(0.00411)
Not part of MSA	0.0253**	-0.00244	0.00900*	0.000456	-0.00514	0.00139	-0.000900	0.00126
	(0.00869)	(0.00237)	(0.00424)	(0.00163)	(0.00477)	(0.00133)	(0.00202)	(0.000889)
Missing MSA	-0.112**	0.0185	0.0261	0.000526	-0.0251	-0.000276	-0.00324	0.000178
	(0.0428)	(0.0140)	(0.0227)	(0.00225)	(0.0265)	(0.000988)	(0.0114)	(0.000589)
Months incarcerated	0.000243*	-0.0000660**	-0.0000784	-0.0000217	0.0000405	-0.0000197*	0.0000333	-0.00000633
	(0.000116)	(0.0000177)	(0.0000434)	(0.0000175)	(0.0000663)	(0.00000916)	(0.0000352)	(0.00000496)
No Supervision	-0.0470**	-0.00592*	-0.0197**	-0.00221	0.0109	0.00197	0.0109	0.00110
	(0.0172)	(0.00236)	(0.00466)	(0.00222)	(0.0124)	(0.00309)	(0.00677)	(0.00218)
Other	-0.0234	0.000276	-0.0120*	-0.00348	-0.00692	0.00193	0.00323	0.00112
	(0.0138)	(0.00440)	(0.00536)	(0.00226)	(0.00863)	(0.00215)	(0.00370)	(0.00165)
Medium	0.0430**	0.00247	-0.00290	0.00322**	0.0204**	-0.000935	0.00574**	0.000431
	(0.00714)	(0.00158)	(0.00301)	(0.00104)	(0.00393)	(0.000896)	(0.00168)	(0.000583)
Med/max	0.147**	0.0175	0.0508**	0.0108	0.0572**	0.00645	0.00829	0.00218
	(0.0240)	(0.0110)	(0.0150)	(0.00734)	(0.0153)	(0.00352)	(0.00578)	(0.00227)
Maximum	0.0339**	-0.0000263	-0.00889*	0.00312	0.0541**	-0.000590	0.0152**	-0.000659
	(0.0117)	(0.00220)	(0.00430)	(0.00181)	(0.00848)	(0.00160)	(0.00407)	(0.000412)

© 2022 Burns ME et al. *JAMA Network Open.*

Jail	-0.0485	-0.00986**	-0.0341**	-0.00273**	-0.0343**	-0.00357**	0.0131	0.0163
	(0.0425)	(0.00188)	(0.00365)	(0.000926)	(0.00378)	(0.000880)	(0.0171)	(0.0171)
Constant	-0.0620**	0.0161**	0.0123	0.00551	-0.0132	0.00520	-0.0136*	0.000192
	(0.0213)	(0.00534)	(0.00921)	(0.00435)	(0.0123)	(0.00273)	(0.00534)	(0.00145)
Observations	16307	16307	16307	16307	16307	16307	16307	16307
R^2	0.079	0.023	0.026	0.010	0.014	0.002	0.006	0.003

*Notes: The coefficients shown reflect the absolute difference in the outcome compared to the reference category for categorical and binary variables. For continuous measures, the coefficient reflects the absolute difference in the outcome for a one-unit increase. The first three rows describe policy variables that characterize the enrollment assistance program: the phase-in period (Jan 2015 – March 2015) with and without a benefit specialist, and a term for the fully implemented period that doesn't differentiate between programs with and without a benefits specialist (>= April 2015). OUD indicates opioid use disorder, SUD indicates substance use disorder, and ED indicates emergency room. For example, relative to the baseline period, the likelihood of any outpatient visit increased by 7.33 percentage points (.0733 * 100) for person-releases exposed to the fully implemented enrollment assistance program. Standard errors in parentheses.* * *p < 0.05,* ** *p < 0.01*

© 2022 Burns ME et al. *JAMA Network Open.*

eTable 5. Unadjusted Outcomes Restricted to First Release per Subject, N=16,307 Releases

Outcome	Baseline, Apr 2014 - Dec 2014 Mean (95% CI)	Enrollment assistance, Jan 2015 - Dec 2016 Mean (95% CI)	P-value
Any outpatient visit	0.166 (0.155, 0.176)	0.242 (0.235, 0.249)	0.000
Outpatient visit with OUD diagnosis	0.007 (0.004, 0.009)	0.014 (0.012, 0.016)	0.000
Outpatient visit with SUD diagnosis	0.027 (0.022, 0.031)	0.037 (0.034, 0.040)	0.000
Medication treatment for OUD	0.003 (0.001, 0.004)	0.007 (0.005, 0.008)	0.000
ED visit	0.056 (0.049, 0.062)	0.062 (0.058, 0.066)	0.111
ED visit for overdose	0.003 (0.001, 0.004)	0.004 (0.003, 0.005)	0.230
Inpatient stay	0.008 (0.005, 0.010)	0.011 (0.009, 0.013)	0.032
Inpatient stay for overdose	0.001 (-0.000, 0.001)	0.002 (0.001, 0.0020	0.048

© 2022 Burns ME et al. *JAMA Network Open.*

eTable 6. ITT Regression Results With Facility Fixed Effects

	(1)	(2)	(3)	(4)	(5)	(6)	(7)	(8)
	**Any outpatient visit**	**Outpatient visit with OUD diagnosis**	**Outpatient visit with SUD diagnosis**	**Medication treatment for OUD**	**ED visit**	**ED visit for overdose**	**Inpatient stay**	**Inpatient stay for overdose**
Phase-in, no benefits specialist	0.0251	0.00125	0.000909	-0.0000723	0.0115	-0.000291	0.00288	0.000704
	(0.0174)	(0.00417)	(0.00787)	(0.00295)	(0.0104)	(0.00248)	(0.00406)	(0.00146)
Phase-in, benefits specialist present	0.0393	-0.00621	-0.000314	-0.00512	0.00687	0.000758	0.000621	0.00312
	(0.0221)	(0.00596)	(0.0108)	(0.00369)	(0.0132)	(0.00381)	(0.00509)	(0.00282)
Full implementation	0.0759**	0.00685**	0.00979**	0.00390**	0.00747	0.00132	0.00339*	0.000975
	(0.00658)	(0.00169)	(0.00292)	(0.00112)	(0.00406)	(0.00102)	(0.00167)	(0.000511)
Female	0.275**	0.0272*	0.0347*	0.00869	0.0588**	-0.00530**	0.0150	-0.00217*
	(0.0331)	(0.0124)	(0.0159)	(0.00759)	(0.0218)	(0.00163)	(0.0107)	(0.00110)
Age	0.00571**	-0.000351**	0.000328**	-0.0000773	0.00137**	0.0000120	0.000429**	-0.00000321
	(0.000313)	(0.0000611)	(0.000125)	(0.0000488)	(0.000188)	(0.0000435)	(0.0000851)	(0.0000233)
Black	-0.0765**	-0.0146**	-0.0259**	-0.00591**	0.00406	-0.00435**	-0.00554**	-0.000861
	(0.00672)	(0.00146)	(0.00254)	(0.00105)	(0.00427)	(0.000967)	(0.00176)	(0.000529)
Other	-0.0720**	-0.0128**	-0.00862	-0.00592**	-0.00122	-0.00540**	-0.00537*	-0.00186**
	(0.0127)	(0.00287)	(0.00635)	(0.00191)	(0.00798)	(0.000753)	(0.00266)	(0.000492)
>= H.S.	0.00432	0.00434**	0.00998**	0.00182	-0.0116**	-0.00232	-0.000738	-0.000199
	(0.00700)	(0.00168)	(0.00285)	(0.00122)	(0.00447)	(0.00121)	(0.00180)	(0.000596)
Missing	0.0195	0.00292	0.00236	0.00230	-0.0144	-0.00595**	-0.00403	-0.00179
	(0.0182)	(0.00567)	(0.00852)	(0.00413)	(0.00982)	(0.00196)	(0.00408)	(0.00197)
Married	-0.0152	0.000106	0.00145	0.000144	-0.00589	-0.000405	0.00172	0.00187
	(0.0109)	(0.00262)	(0.00485)	(0.00180)	(0.00613)	(0.00145)	(0.00294)	(0.00133)
Other	0.00281	0.00112	0.00488	0.00328	0.0284*	0.00252	0.00930	0.00540
	(0.0205)	(0.00644)	(0.00975)	(0.00503)	(0.0128)	(0.00323)	(0.00597)	(0.00342)
Not part of MSA	0.0239**	-0.00245	0.00872*	-0.0000341	-0.00656	0.000302	-0.00156	0.000985
	(0.00847)	(0.00235)	(0.00414)	(0.00163)	(0.00472)	(0.00134)	(0.00199)	(0.000891)
Missing	-0.107**	0.0165	0.0230	0.0000574	-0.0232	0.000595	-0.00411	0.000491
	(0.0409)	(0.0136)	(0.0221)	(0.00225)	(0.0260)	(0.00106)	(0.0109)	(0.000572)
Months incarcerated	0.000269*	-0.0000356*	-0.0000354	-0.0000113	-0.0000186	-0.0000283**	0.0000493	-0.00000882
	(0.000119)	(0.0000159)	(0.0000433)	(0.0000177)	(0.0000690)	(0.0000101)	(0.0000366)	(0.00000516)
No Supervision	-0.0521**	-0.00723**	-0.0202**	-0.00338	0.00426	0.000776	0.0119	0.00219
	(0.0153)	(0.00196)	(0.00412)	(0.00180)	(0.0110)	(0.00297)	(0.00615)	(0.00238)
Other	-0.0339*	-0.00878**	-0.0236**	-0.00759**	-0.00512	0.000866	0.00203	0.000524
	(0.0137)	(0.00334)	(0.00490)	(0.00243)	(0.00855)	(0.00226)	(0.00431)	(0.00156)
Constant	-0.109**	0.0193**	-0.0183	0.0111*	-0.0214	0.00766*	-0.00817	-0.000180
	(0.0255)	(0.00583)	(0.0118)	(0.00477)	(0.0153)	(0.00385)	(0.00617)	(0.00157)
Observations	18265	18265	18265	18265	18265	18265	18265	18265
R^2	0.086	0.024	0.029	0.011	0.017	0.012	0.012	0.023

© 2022 Burns ME et al. *JAMA Network Open.*

*Notes: The coefficients shown reflect the absolute difference in the outcome compared to the reference category for categorical and binary variables. For continuous measures, the coefficient reflects the absolute difference in the outcome for a one-unit increase. The first three rows describe policy variables that characterize the enrollment assistance program: the phase-in period (Jan 2015 – March 2015) with and without a benefit specialist, and the fully implemented period that doesn't differentiate between programs with and without a benefits specialist (>= April 2015). OUD indicates opioid use disorder, SUD indicates substance use disorder, and ED indicates emergency room. For example, relative to the baseline period, the likelihood of any outpatient visit increased by 7.59 percentage points (.0759 * 100) for person-releases exposed to the fully implemented enrollment assistance program. Standard errors in parentheses.* * $p < 0.05$, ** $p < 0.01$.

© 2022 Burns ME et al. *JAMA Network Open.*

eReferences.

1. Andrews CM, Pollack HA, Abraham AJ, et al. Medicaid coverage in substance use disorder treatment after the affordable care act. *J Subst Abuse Treat.* 2019;102:1-7. doi: 10.1016/j.jsat.2019.04.002
2. Chandler RK, Fletcher BW, Volkow ND. Treating Drug Abuse and Addiction in the Criminal Justice System Improving Public Health and Safety. JAMA. 2009;301(2):183-190. doi: 10.1001/jama.2008.976
3. Carson EA . Prisoners in 2019. Washington, D.C. : Bureau of Justice Statistics October 2020. NCJ 252156.
4. Donohue JM, Jarlenski MP, Kim JY, Tang L, Ahrens K, Allen L., et al. Use of Medications for Treatment of Opioid Use Disorder among US Medicaid Enrollees in 11 states, 2014-2018." JAMA. In Press.
5. Western B, Smith N. Formerly Incarcerated Parents and Their Children. Demography. 2018;55(3):823-847. doi: 10.1007/s13524-018-0677-4
6. Lee DS, Moreira MJ, McCrary J, Porter J. Valid t-ratio Inference for IV. Princeton University unpublished manuscript.

© 2022 Burns ME et al. *JAMA Network Open.*
